# Assessment of the Suitability of Coke Material for Proppants in the Hydraulic Fracturing of Coals

**DOI:** 10.3390/ma16114083

**Published:** 2023-05-30

**Authors:** Tomasz Suponik, Krzysztof Labus, Rafał Morga

**Affiliations:** Faculty of Mining, Safety Engineering and Industrial Automation, Silesian University of Technology, Akademicka 2, 44-100 Gliwice, Poland

**Keywords:** hydraulic fracturing, proppant, coke, coal bed methane

## Abstract

To enhance the extraction of methane gas from coal beds, hydraulic fracturing technology is used. However, stimulation operations in soft rocks, such as coal beds, are associated with technical problems related mainly to the embedment phenomenon. Therefore, the concept of a novel coke-based proppant was introduced. The purpose of the study was to identify the source coke material for further processing to obtain a proppant. Twenty coke materials differing in type, grain size, and production method from five coking plants were tested. The values of the following parameters were determined for the initial coke: micum index 40; micum index 10; coke reactivity index; coke strength after reaction; and ash content. The coke was modified by crushing and mechanical classification, and the 3–1 mm class was obtained. This was enriched in heavy liquid with a density of 1.35 g/cm^3^. The crush resistance index and Roga index, which were selected as key strength parameters, and the ash content were determined for the lighter fraction. The most promising modified coke materials with the best strength properties were obtained from the coarse-grained (fraction 25–80 mm and greater) blast furnace and foundry coke. They had crush resistance index and Roga index values of at least 44% and at least 96%, respectively, and contained less than 9% ash. After assessing the suitability of coke material for proppants in the hydraulic fracturing of coal, further research will be needed to develop a technology to produce proppants with parameters compliant with the PN-EN ISO 13503-2:2010 standard.

## 1. Introduction

Coal bed methane is natural gas (NG) found in coal deposits. During the formation of coal, large amounts of this gas are generated, and their part is trapped by the coal matter. Coal has a huge internal surface area; therefore, it can store considerable volumes of methane, more than conventional porous rocks.

As the coal bed methane is held in place by water pressure, to extract it, the water pressure should be reduced by pumping out the fluid through wells, which enables the release of natural gas from the coal. The gas should then be separated from the water and transported to compression plants.

To improve methane gas extraction, hydraulic fracturing technology (HF) has been used since the 1970s [1]. HF consists of injecting a fracturing fluid (FF), under high pressure, into a coal seam to propagate existing fractures and induce a new artificial fracture network. This ensures hydraulic conductivity in the created fractures to achieve a continuous high gas production rate. When the fracture network is induced, in most cases, a propping agent—proppant, commonly sieved sand or ceramic spheres [2], added to the injected fluid—prevents fracture closure after the release of pressure from the injected fracturing fluid once the HF operation is completed. The remaining proppant grains in the fracture prevent it from fully closing and losing hydraulic conductivity. As a result, the propped fractures constitute highly permeable arteries that enable communication between the well and the reservoir [3]. This facilitates the removal of water and extraction of the gas.

**Figure 1:**
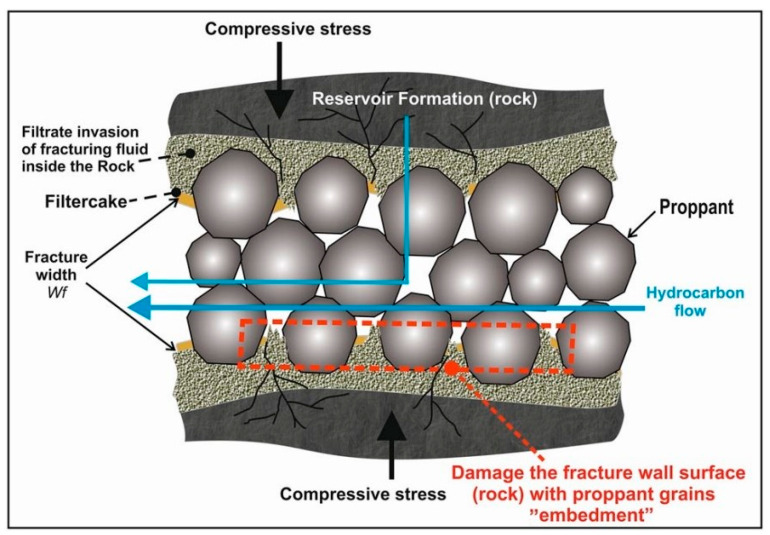
Embedment and other phenomena that affect the surface of the fracture face during hydraulic fracturing [12].

HF technology in the oil and gas industry has long reached the application phase [4,5,6,7,8,9,10,11]. However, hydraulic fracturing in soft rocks, such as coal beds, is associated with technical problems related mainly to the embedment process, which reduces the fracture conductivity (Figure 1). On the other hand, the fracturing operations will take place at depths generally not exceeding 1500 m, which does not require the use of high-strength proppants. Therefore, it is necessary to develop innovative proppant materials that will be suitable for these specific conditions.

Some solutions have already been proposed, an example of which is an ultra-lightweight proppant (ULWP) for the hydraulic fracturing of hydrocarbon deposits and non-hydrocarbon formations, in particular, coal seams, used to increase methane production [13]. This ultra-light backfill, as described, consists of a base material partially covered with a protective coating or hardened. The basic material can be ground nut shells, quartz, glass, sand, or silicates. The particle size of this material is from 12/20 mesh (1.680/0.841 mm) to 40/70 mesh (0.400/0.210 mm). Such small particles are better suspended in the low-viscosity fracturing fluid and easily move in narrow natural fissures.

Although proppant use has been common in deep reservoir fracturing, its use in soft rocks, such as coal, should be carefully investigated [14]. Typical proppants are prone to the embedment phenomenon, and, therefore, are not suitable for soft coal seams. Furthermore, their composition does not correspond to the chemical composition of coal seams, and the proppant used wears the surface of the fracture and reduces its width and conductivity.

The main challenges related to the properties and parameters of proppants for fracking operations in coals are the following [15]:

1. Compressive strength of 13.8 MPa, at which over 90% of grains remain undestroyed;
2. Proppant pack conductivity for 2% KCl solution is higher than 13 × 10^−14^ m^2^·m;
3. Dimensionless fracture conductivity is higher than 40 [-];
4. The optimal transport and settling of proppant in the created fracture;
5. Possibility to control the quality of filling the fracture with proppant;
6. Compatibility of the proppant material with the formation rock and fracking fluid;
7. Maintaining a fracture system of desired conductivity inside the coal rock;
8. Limitation of the embedment phenomenon, consisting of (A) minimizing the depth of indentation of the proppant grains into the fracture wall (carbon rock) to a value not greater than 20% of the average diameter of the grains and (B) maintaining the surface of the fracture wall with less than 35% damage;
9. Reduction in the fracture permeability damage during coal seam demethanization;
10. Reduction in water and material consumption.

Given the desired proppant properties stated above, we developed the concept of a novel, innovative, coke-based proppant:

1. The chemical affinity of coke proppant to coal facilitates underground coal gasification (UCG), or the extraction of demethanized coals as energy carriers or for use in the raw material chemical industry;
2. The considerable porosity and permeability of the proppant allow for more intensive gas migration. The total porosity of the coke proppant can be as high as 50%, with effective porosity reaching 15%. This facilitates gas migration in the propped fracture as well as through the coke proppant itself, unlike most traditional proppants. Gas migration through coke proppant grains may be maintained even when they are partially embedded in the coal rock surface;
3. The porous nature of the coke proppant allows it to be saturated with chemicals designed to control the viscosity of the fracking fluid after stimulation;
4. The structure of the coke proppant limits its embedment. Due to its roughness, it supports the fracture wall at multiple points and reduces embedment compared to proppants of a smoother grain surface, such as treated sand, ceramics, and resin-coated proppants;
5. The low density makes it easy to suspend the proppant in the fracturing fluid and pump it easily into the fractures. The bulk density of the coke material (e.g., coke breeze) can reach 0.57 g/cm^3^, which is lower than for typical frac sand −1.49 g/cm^3^ (for 40/70 mesh) and even ultra-lightweight proppants (ULWP), e.g., −0.66 g/cm^3^ (for 30/80 mesh). The low bulk and specific densities of coke proppants allow them to be used more efficiently with low-viscous foams and energized fracking fluids. It also facilitates the injection of large volumes of proppant from a specific well into the distant parts of the fractures [16].

These characteristics indicate that coke may be a useful material for the production of a proppant for the hydraulic fracturing of coals.

Coke is a material obtained from the pyrolysis of hard coal carried out at a temperature of approximately 1000 °C. Its parameters depend mainly on the petrographic composition and the coking properties of the coal mixture [17,18,19,20,21,22]. The quality of coke is estimated by determining the content of ash, moisture, and volatile matter, as well as the elemental composition [17,23]. Furthermore, when coke is used in the classic blast furnace process, its mechanical properties, such as strength, abrasiveness, and coke reactivity to CO_2_, are assessed, comprising the coke reactivity index (CRI) and coke strength after reaction (CSR) [23,24]. Coke that achieves a low CRI value and a high CSR value is highly valued, especially because it has higher mechanical strength and better gas permeability in metal production processes.

The aim of this preliminary study was to identify the most promising source of coke material for further processing in order to obtain a suitable coke proppant. Due to pending patent proceedings, the authors cannot provide any details of the processing procedures or the final properties of the proppants.

## 2. Materials and Methods

### 2.1. Materials

Twenty coke samples that differed in type, grain size, origin, and method of production were the initial material (Table 1). They were obtained from five coking plants located in southern Poland: “Częstochowa Nowa” in Częstochowa; “Zdzieszowice” in Zdzieszowice; “Przyjaźń” in Dąbrowa Górnicza; “Jadwiga” in Zabrze; and “Radlin” in Radlin.

### 2.2. Methods

#### 2.2.1. Analytical procedures performed on the initial coke materials

**Figure 2:**
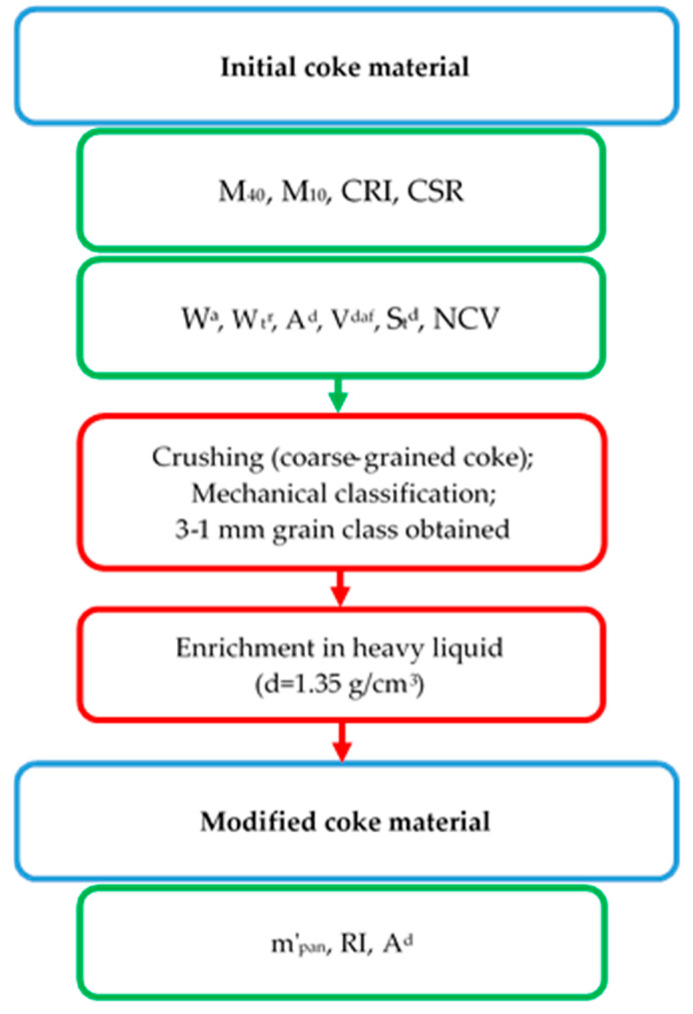
Processing and analytical procedures performed in the study.

The following parameters of the initial cokes were analyzed (Figure 2; Table 2), which characterize their properties and may determine their suitability for use as proppants.

For micum index 40 (M_40_) and micum index 10 (M_10_), the drum method according to [25] was applied to assess the mechanical properties of the coke material, including its strength and abrasiveness (Table 2).

Micum index 40 (M_40_), also known as mechanical strength, is the percentage of the coke residue above the 40 mm sieve size (Equation (1):

(1) $$M_{40}=\frac{m_{2}}{m_{1}}100\%$$

where

*m*_1_—coke mass before tumbling with grain size > 40 mm, g;
*m*_2_—coke mass after tumbling with grain size > 40 mm (residue in a sieve with a mesh size of 40 mm), g;

Micum index 10 (M_10_), referred to as abrasiveness, is the percentage of the coke residue below the 10 mm sieve size (Equation (2)):

(2) $$M_{10}=\frac{{m_{2}}^{\prime }}{{m_{1}}^{\prime }}100\%$$

where

*m*_1_′—coke mass before tumbling, g;
*m*_2_′—the loss after tumbling through a 10 mm mesh sieve, g;

CRI and CSR indices were determined via a widely used method developed by Nippon Steel Corporation [26] (Table 2).

Coke reactivity index (CRI), being the measure of coke reactivity, is the percentage mass loss of the coke sample treated with CO_2_ (Equation (3):

(3) $$\mathrm{CRI}=\frac{{m_{1}}^{\prime \prime }-{m_{2}}^{\prime \prime }}{{m_{1}}^{\prime \prime }}100\%$$

where

*m*_1_″—coke mass before determination of reactivity, g
*m*_2_″—coke mass after determination of reactivity, g;

Coke strength after reaction with CO_2_ (CSR) is the mass of coke that remains on a 10 mm square mesh sieve after mechanical treatment in a standardized rotary drum related to the mass of the sample remaining after the determination of reactivity (Equation (4)):

(4) $$\mathrm{CSR}=\frac{{m_{3}}^{\prime \prime \prime }}{{m_{2}}^{\prime \prime \prime }}100\%$$

where

*m*_2_‴—coke mass after determination of reactivity before tumbling, g;
*m*_3_‴—coke mass after determination of reactivity and with a grain size greater than 10 mm after tumbling, g;

Ash content (A^d^) was determined according to [27] (Table 2). The value of this parameter is important as an increase in the ash content reduces the mechanical strength of coke [23]. 

Furthermore, the following parameters were determined for a better understanding of the basic properties of the initial coke materials (Table 2): moisture content (total, W^a^, and in the analytical sample, W_t_^r^) [28,29], volatile matter content (V^daf^) [30], total sulfur content (S_t_^d^)—measured using a LECO SC 132 analyzer—and net calorific value (NCV) [31].

**Table 2:** Parameters of the coke materials determined in the study.

Parameter	Standard/Method
Micum index 40 (M_40_)	PN-C-04305:1998 [25]
Micum index 10 (M_10_)	PN-C-04305:1998 [25]
Coke reactivity index (CRI)	ISO 18894:2006 [26]
Coke strength after reaction with CO_2_ (CSR)	ISO 18894:2006 [26]
Ash content (A^d^)	PN-ISO 1171:2002 [27]
Total moisture content (W^a^)	PN-ISO 579:2002 [28]
Moisture content in the analytical sample (W_t_^r^)	PN-ISO 687:2005 [29]
Volatile matter content (V^daf^)	PN-G-04516:1998 [30]
Total sulfur content (S_t_^d^)	Combustion of samples at 1350 °C using the LECO SC 132 analyzer
Net calorific value (NCV)	PN-ISO 1928:2020-05 [31]
Crush resistance index (m′_pan_)	PN-EN ISO 13503-2:2010 [32]
Roga index (RI)	PN-ISO 15585:2009 [33]

#### 2.2.2. Modification of the initial coke materials

To make an in-depth preliminary assessment of their suitability for use as proppants, the initial coke materials were subjected to modification (Figure 2). The method of modification depended on the grain size of the cokes. For coarse-grained coke (samples I, III, V, VII, IX, X, XI, XII, XIV, XV, XVI, and XX), it consisted of successive crushing in two jaw crushers, the first with a gap of 20 mm (the average grain size after crushing varied from 20 mm to 8 mm), the second with a gap of 6 mm (the average grain size after crushing varied from 6 to 0.5 mm), and mechanical classification using sieves with a mesh size of 1 and 3 mm. For coke breeze (samples II, IV, VI, VIII, XIII, XVII, XVIII, XIX), it involved mechanical classification using sieves of 1 and 3 mm mesh size only.

The resulting materials in class 3–1 mm were subjected to a simplified densiometric analysis [34] in a heavy liquid with a density of 1.35 g/cm^3^ (calcium chloride solution was used). The lighter density fraction was dried in an oven at 105 °C to a constant sample weight.

#### 2.2.3. Analytical procedures performed on the modified coke materials

The lighter density fraction was further tested to determine technological parameters (Table 2).

A crush resistance test of the modified coke material (m′_pan_) was performed [32]. The test is useful for determining and comparing the strength of proppants. It was carried out on samples that were sieved so that all of the particles tested were within the specified size range. The amount of material crushed under a stress of 13.8 MPa was measured.

Mechanical strength according to the modified Roga method (RI_mod_) [33,35] was also analyzed. The coke material was weighed and subjected to three five-minute tumblings at 50 rpm in a Roga drum filled with 100 g of coke and 1000 g of steel balls of 12 mm diameter. The operation of sieving through a 1 mm square mesh sieve and weighing was repeated after each cycle (Equation (5). The tests were carried out twice. The arithmetic mean was taken as a result.

(5) $$\mathrm{RI}=\frac{100}{3Q}\left[\frac{a+d}{2}+b+c\right]$$

where

*Q*—weight of the sample after coking (before the first sieving), g;
*a*—weight of the sample on the sieve before the first tumbling, g; *Q* = *a* = 100 g;
*b*—weight of the sample on the sieve after the first tumbling, g;
*c*—weight of the sample on the sieve after the second tumbling, g;
*d*—mass of the sample on the sieve after the third tumbling, g.

The ash content (A^d^) in the modified coke materials was measured again [27].

## 3. Results and Discussion

### 3.1. Properties of the initial and modified coke materials

**Figure 3:**
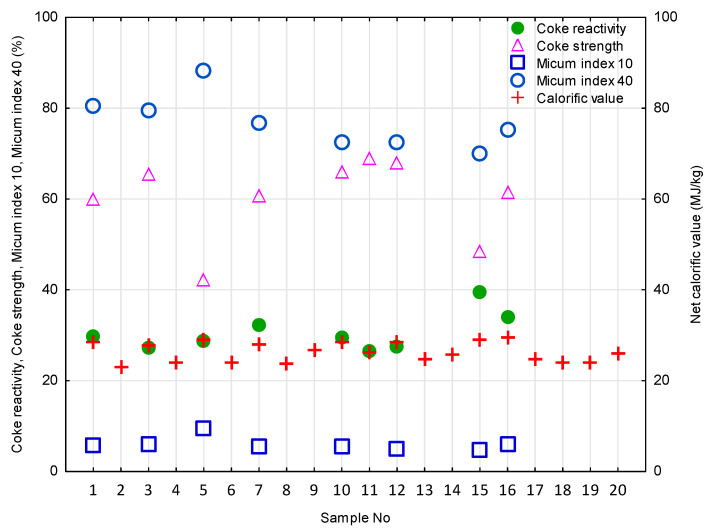
Strength parameters and net calorific value of the coke materials tested.

**Figure 4:**
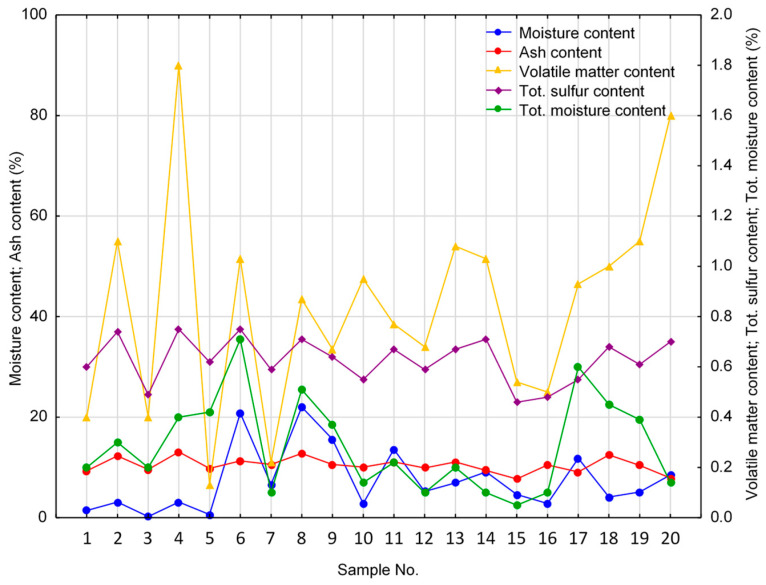
Results of the technical analysis of the coke materials tested.

Micum index 40 (M_40_) for the initial coke material was at least 70 (mostly exceeding 75), while micum index 10 (M_10_) remained below 10 (Figure 3). This shows the relatively high strength and resistance to breakage and abrasion of the studied cokes, adequately meeting the European requirements [17,19]. The coke reactivity index (CRI) ranged between 27.3 and 39.4, with most values being below 30, while coke strength after the reaction (CSR) varied from 42.2 to 69.0, usually exceeding 60 (Figure 3). CRI<30 and CSR>60 are the required values for high-quality blast furnace cokes [17,18,19]. Moreover, CSR>60 indicates the high mechanical strength of the studied material [17]. Foundry coke (sample V) was found to have an exceptionally low CSR value (42.2). The ash content (A^d^) ranged from 7.7% to 13.1%, at an average value of 10.5% (Figure 4), being typical for different kinds of cokes, including the European blast furnace cokes [17,19].

The other parameters of the initial coke material, such as total moisture content (W^a^), the moisture content in the analytical sample (W_t_^r^), volatile matter content (V^daf^), total sulfur content (S_t_^d^), and net calorific value (NCV), are given in Figure 3 and Figure 4.

The feasibility of enriching coke materials to improve their strength depends not only on their initial ash content but also on their homogeneity. If the material is isotropic, enrichment is difficult or impossible. Only the coke materials that are inhomogeneous in terms of their mineral content are enrichable. In this study, it was assumed that coke materials of the highest strength parameters and with an ash content (A^d^) <9% would be selected for further detailed work covering technological (processing) operations to obtain the best proppant for the hydraulic fracturing of coals. Therefore, the grain class of 3–1 mm obtained by the mechanical classification of each coke material was enriched in heavy liquid with a density of 1.35 g/cm^3^, and a set of parameters (m′_pan_, RI and A^d^) was determined for the lighter fraction.

**Figure 5:**
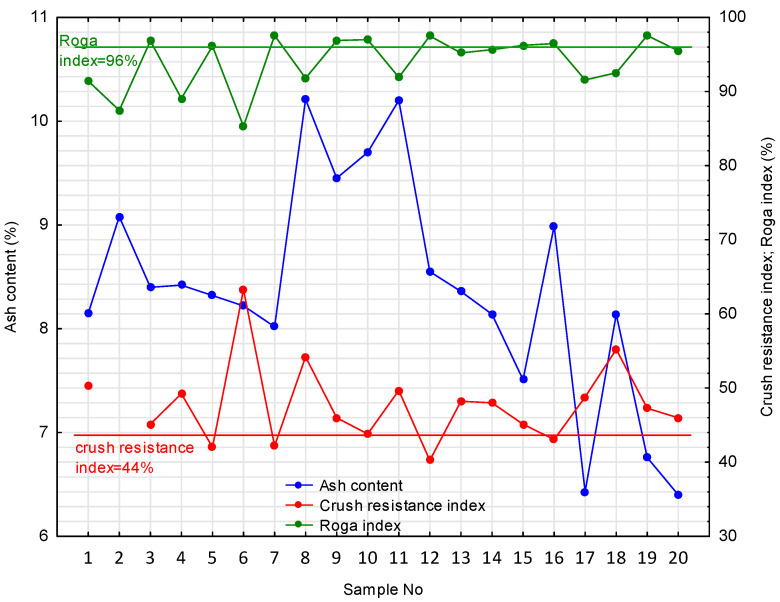
Technological parameters of the modified coke materials.

The modified coke materials were characterized by a varied crush resistance index (m′_pan_) (40.25–63.28%) and Roga index (85.30–97.55), reflecting their different strengths (Figure 5).

The ash contents were significantly lower than in the initial coke materials, with most samples meeting the A^d^<9% criterion (Figure 5). The average ash contents in the light fraction for coarse-grain coke and coke breeze were 8.49 and 8.08%, which means that the difference between them was small, and, in the heavy fraction, they were 16.70 and 19.85%, respectively, which proves that in the coke breeze, there was 3.15 p.p. (percentage point) more mineral matter than in coarse coke. The average ash contents in the combined density fractions of coarse-grained coke and coke breeze are 11.11% and 12.85%. The results indicate a significant loss of mineral substance in the light fraction of the coke materials, which contributed to the improvement in the strength parameters of the modified cokes. Samples II and VIII-XI were difficult to enrich. They represented different types of coke, but they were usually fine-grained and were obtained from different coking plants (Table 1). Although the ash content was reduced with reference to the initial material, it remained above the desired level (Figure 5).

### 3.2. Selection of the Most Promising Material for the Final Processing to Obtain a Coke Proppant

As mentioned earlier, parameters that have a strong influence on the decision on the suitability of coke materials for use as proppants are coke strength indicators such as M_40_, M_10_, CRI, CSR, m′_pan_, and RI. The parameters m′_pan_ and RI were chosen as key parameters because the tests were carried out for modified samples, and this is the kind of coke material that is to be evaluated as a potential raw material for proppant production.

From this point of view, the studied coke materials were divided into three groups: cokes of the highest, medium, and lowest strength. The following values of the technological parameters were used as a criterion for the division:

1. <44%/44–49%/>49% (highest/average/lowest strength)—for the crush resistance of the coke material, m′_pan_;
2. >98% ±2%/96–92% ±2%/<90% ±2% (highest/average/lowest strength) for RI, that is, for mechanical strength according to the modified Roga method.

Consequently, on the basis of the criteria adopted, the coke materials were divided into three groups:

1. Cokes of the highest strength—samples V, VII, X, XII, XVI;
2. Cokes of medium strength—samples III, IX, XIII, XIV, XV, XVII, XIX, XX;
3. Cokes of the lowest strength—samples I, IV, VI, VIII, XI, XVIII.

The values of the micum index 40 (M_40_) for the coke materials in the highest strength group ranged from 72.4% (sample XII) to 88.2% (sample V), while the values of the micum index 10 (M_10_) ranged from 5.0% (sample XII) to 9.6% (sample V) (Figure 3). All materials in this group had similar coke reactivity to CO_2_ (CRI). Sample V was characterized by the lowest strength after reaction (CSR), 42.2%, compared to an average of 64.1% for the others in this group (Figure 3).

High values of M_40_ and CSR and low values of M_10_ and CRI were also characteristic of samples I and III (Figure 3). This predisposed them to the group of cokes with the highest strength. However, the values of the m′_pan_ and RI determined after the modification of the coke materials were relatively low. This caused them to be placed in the group of samples with the lowest and medium strength, respectively.

The parameters M_40_, M_10_, CRI, and CSR were not investigated for fuel coke and coke breeze, as they are only determined for large grain classes. Coke breeze, as a waste material from the production of foundry coke, blast furnace coke, or fuel coke, could, by definition, be an interesting alternative for the production of proppants. As a waste material, it is inexpensive, and its grain size range from 10 to 0 mm means that it does not require grinding as part of the coke material modification process. By omitting this energy-consuming operation, production costs could be reduced. However, all of the breeze cokes, due to their low values of the m′_pan_ and RI parameters, were classified into the group of medium- and lowest-strength cokes.

It should be added at this point that sample II, i.e., the coke breeze from the production of blast furnace coke in the 3–0 mm class, contained only 1.76% of the 3–1 mm grain class and 98.24% of the 1–0 mm grain class. The negligible amount of grain class 3–1 mm and the potential for its use made it impossible (owing to the unprofitability of future production) to process it. Therefore, this sample was rejected. The analyses carried out on the RI and ash content of the enrichment product (Figure 5) further indicate that this material has a low potential for use as a proppant.

In summary, it can be concluded that on the basis of parameters M_40_ and M_10_, it is not possible to clearly determine whether coke will be suitable for the preparation of the proppant. It can only be said that their values should be at least 70% and at most 10%, respectively. To initially select the best cokes for proppant preparation, further tests should be carried out to determine the strength parameters of crushed coke to the 3–1 mm class after its initial modification consisting of the removal of heavy coke fractions containing an increased content of mineral substance expressed as ash content. These parameters include crush resistance (m′_pan_) and mechanical strength, according to the modified Roga method (RI_mod_). These parameters should be at least 44% and at least 96%, respectively.

As this is the first time that the raw material for the coke-based proppant has been sought, it is not possible to compare the properties of the best materials found in this study with those of any other cokes investigated for a similar purpose.

### 3.3. The role of fracture conductivity

It should also be noted here that one of the key factors that affects the productivity of coal bed gas wells is fracture conductivity [36]. CBM reservoirs, due to their low values of permeability, Young’s modulus, relatively low pressure, and high Poisson ratio are significantly different from those of conventional hydrocarbon deposits. It has been found that fracture conductivity is related to reservoir properties, closure pressure, proppant properties, and concentration, as well as fracturing fluid composition and rheology. The conductivity increases with the diameter of the proppant particles and the number of proppant layers, whereas it decreases with the decreasing closing pressure. At the same closing pressure, the conductivity of a multi-layer proppant pack is greater than the conductivity of a single-layer pack. In the early stages of fracturing, with fine-grained proppants, the extent of the fracture may be longer. For the final stage of fracturing, injected coarser proppants are able to improve near-wellbore conductivity [37]. The complicated issues described above mean that for each type of coal and the proppant and fracturing fluid used, hydraulic fracture conductivity tests should be performed.

## 4. Conclusions

This is the first time that the source material for coke-based proppants has been identified. On the basis of the tests carried out, the following conclusions can be drawn:

1. The preliminary assessment of the suitability of the source coke material for the final processing is based on a set of tests performed on the initial coke and the 3–1 mm grain fraction enriched in a heavy liquid with a density of 1.35 g/cm^3^. For the initial coke, these include the strength parameters M_40_, M_10_, CRI, CSR, and ash content (A^d^). For the modified cokes, they comprise m′_pan_ and RI, which were selected as key strength parameters, and A^d^.
2. The most promising modified coke materials with the best strength properties had m′_pan_ and RI values of at least 44% and at least 96%, respectively, and contained less than 9% ash. They were obtained from a coarse-grained (fraction 25–80 mm or larger) blast furnace and foundry coke. For these materials, the values of the individual parameters are as follows: M_40_ > 72.4%, M_10_ < 9.6%, CRI < 34.1%, and CSR > 42.2%.
3. Further work on the highest strength coke materials described in this article will consist of developing a technology for the production of proppants in order to obtain a product with parameters compliant with the PN-EN ISO 13503-2:2010 standard. They will consist, in particular, of the selection of effective and environmentally friendly methods of crushing, shaping, and enriching coke materials.

## Figures and Tables

**Table 1:** Type, grain size, and production method of the tested coke materials.

Sample	Producer	Type	Method of Filling the Chambers and Quenching the Coke; Coking Time	Grain Size, mm
I	Coking plant 1	Blast furnace coke	Hopper system; wet quenching; 16:30 h	25–80
II	Coking plant 1	Coke dust (coke breeze) after production of blast furnace coke nr I	Hopper system; wet quenching; 16:30 h	0–3
III	Coking plant 1	Blast furnace coke	Hopper system; dry quenching; 16:30 h	25–80
IV	Coking plant 1	Coke dust (coke breeze) after production of dry-quenched blast furnace coke nr III	Hopper system; dry quenching; 16:30 h	0–10
V	Coking plant 2	Foundry coke	Stamper system; wet quenching; 29:00 h	>80
VI	Coking plant 2	Coke dust (coke breeze) after production of blast furnace coke nr V	Stamper system; wet quenching; 29:00 h	0–10
VII	Coking plant 2	Blast furnace coke	Stamper system; wet quenching; 26:00 h	25–80
VIII	Coking plant 2	Coke dust (coke breeze) after production of blast furnace coke nr VII	Stamper system; wet quenching; 26:00 h	0–10
IX	Coking plant 2	Fuel coke	Stamper system; wet quenching; 26:00 h	10–25
X	Coking plant 3	Blast furnace coke stabilized	Stamper system; wet quenching; 21:00 h	25–80
XI	Coking plant 3	Fuel coke	Stamper system; wet quenching; 21:00 h	10–30
XII	Coking plant 4	Blast furnace coke	Stamper system; wet quenching; 41:09 h	25–80
XIII	Coking plant 4	Coke dust (coke breeze) after production of blast furnace coke nr XII	Stamper system; wet quenching; 41:09 h	0–10
XIV	Coking plant 4	Fuel coke	Stamper system; wet quenching; 41:09 h	10–25
XV	Coking plant 5	Blast furnace coke	Stamper system; wet quenching; 20:35 h	40–80
XVI	Coking plant 5	Foundry coke	Stamper system; wet quenching; 25:39 h	60–100
XVII	Coking plant 5	Coke dust (coke breeze) after production of foundry coke nr XVI	Stamper system; wet quenching; 25:39 h	0–10
XVIII	Coking plant 1	Coke dust (coke breeze) after production of blast furnace coke nr I	Hopper system; wet quenching; 24:30 h	0–10
XIX	Coking plant 5	Coke dust (coke breeze) after production of blast furnace coke nr XV	Stamper system; wet quenching; 20:35 h	0–10
XX	Coking plant 5	Fuel coke	Stamper system; wet quenching; 26:24 h	10–20

## Data Availability

The data presented in this study are available on request from the corresponding authors.
